# A Postscript on Sunday Games

**Published:** 1921-03-19

**Authors:** 


**THE PUBLIC WELL-BEING**—*(continued).*

### A Postscript on Sunday Games.

A few weeks ago an article appeared in these columns urging, on moral as well as physical grounds, the importance of opening public parks and playgrounds on Sundays, for the playing of games, and pointing out that the demand of hundreds of thousands of young people of both sexes for this modest privilege was becoming irresistible. The writer was, of course, aware that a sabbatarianism continues to flourish within narrow limits, and that, in the regard of the strict sabbatarian, all Sunday activities beyond the walk to and from church and the eating of meals continue to be a profanation. He was a little surprised, however, in view of the healthy differences of opinion that exist among good Christians on this matter, to receive a letter from a scandalised sabbatarian accusing him of being an

**THE PUBLIC WELL-BEING**—(*continued*).

atheist and hinting quite intelligibly at the torments awaiting him in the hereafter. If such is to be his fate, he will share it with a large number of orthodox parsons, some of whom not only give "cinema shows" on Sunday but play games on the same day.

If our accuser keeps up to date as a reader of non-Sunday newspapers he will have observed that Dean Inge this week qualified shamelessly for a letter of excommunication. He made a sensational start in an article in the *Evening Standard* by quoting from the Cambridge manuscript of the New Testament, which ranks fifth in importance among the manuscripts containing the Greek text. In this manuscript, he reminded us, there is a curious insertion after Luke vi., 4. It runs as follows: "On the same day He saw a man working on the Sabbath day, and said to him: Man, if thou knowest what thou doest, thou art blessed; but if thou knowest not, thou art cursed, and a transgressor of the law." We are neither able nor eager to enter into a theological controversy on Sunday observance, but this passage certainly suggests a sound and not irreverent point of view for all who entertain deep religious convictions about the Sabbath to adopt towards this question. In our view, Dean Inge sums up in a single sentence the attitude of reasonable Christians. "Sunday," he said, "ought to be a day for tuning up the health of soul, mind and body together, and it does not at present fulfil this function, because it is treated simply as a day of idleness." What we recently tried to point out was that the street-corner loafing that is a vicious commonplace of the English Sunday in all the large towns is destructive of "the health of soul, mind and body"; and it was a recognition of this which prompted an appeal to the Ministry of Health to find a healthy outlet for dangerous idleness by encouraging local authorities everywhere to throw open recreation grounds on Sundays. If Dr. Addison responds to our appeal, he will no doubt be called an atheist in his turn, but he has been called so many names in recent months that an addition to the list will scarcely disturb his sleep.

